# Placebo use and outcome quality: A protocol for systematic review and meta-analysis

**DOI:** 10.1097/MD.0000000000022915

**Published:** 2020-10-30

**Authors:** Juliana C. R. Alves da Silva, Kesley Pablo Morais de Azevedo, Cecília Menezes Farinasso, Dayde Lane Mendonça da Silva, Cristine Stefani, Ana Cláudia Morais Godoy Figueiredo, Patrícia Medeiros de Souza, Grasiela Piuvezam, Helaine Carneiro Capucho

**Affiliations:** a University of Brasilia, University Campus Darcy Ribeiro Asa Norte, Brasília, Distrito Federal; b Department of Public Health, Postgraduate Program in Public Health, Federal University of Rio Grande do Norte, University Campus Lagoa Nova, Natal, Rio Grande do Norte; c Ministry of Health, Esplanade of Ministries, Brasília, Distrito Federal, Brazil.

**Keywords:** clinical trials, new drugs, new pharmaceutical products, placebo, systematic review

## Abstract

**Background:**

The Pharmaceutical industry sponsorship, research outcome and quality has been already evaluated for clinical trials in order to analyze if this kind of sponsorship affects the results of clinical trials. In this sense, this study has the aim to investigate whether placebo use allows positive outcomes regarding efficacy and safety compared to synthetic medicines.

**Methods:**

We designed and registered a study protocol for a systematic review for methodology data. We will only randomized clinical trials that use placebo as comparator. The main outcome will be the evaluation of placebo use regarding the tendency for positive results (efficacy and security) when comparing to synthetic medicines. PubMed, Cochrane, LILACS (BVS), Web of Science, Scopus, and Excerpta Medica dataBASE (EMBASE) databases will be searched. Gray literature will be identified through the databases Proquest (Dissertation and Theses), OpenGrey and Google Scholar. Two review authors will independently assess trial quality and will extract data in accordance with standard Cochrane methodology. If necessary, we will also contact authors for additional information. The Cochrane Collaboration's risk of bias tool will be used. If feasible, it means homogenous data, we will conduct random effects meta-analysis. Subgroup analyses will be conducted for different justifications for placebo use and for studies sponsored/not sponsored by the pharmaceutical industry.

**Results:**

Our present findings will indicate the effects of placebo use as comparator regarding efficacy and safety of the oral synthetic medicines.

**Discussion:**

This systematic review will identify, summarize, and analyze if there is a trend for positive efficacy and safety results for synthetic medicines in clinical trials when compared with placebo and if the justification for placebo use is considered ethically acceptable.

**Systematic review registration:**

PROSPERO CRD42018110829

## Abstract 2

Supplemental Digital Content is available in the text

## 1 Introduction

To introduce a drug product on the market, it must first be established that this product is safe and effective for the treatment of the disease to which it has been proposed. Besides, to verify these requirements, clinical studies in humans are carried out in several phases.^[1]^

The safety assessment is related to the understanding that every medicine use presents risks, therefore, it is necessary to evaluate the risk-benefits of its use. The efficacy, however, considers the medicine's ability to achieve the expected diagnostic, prophylaxis and/or cure results for that particular molecule. In this sense, the safety and efficacy profile is based on a detailed assessment of the benefits related to the risks.^[1–2]^

The word placebo was first used, in the early 1960s, to refer to a product that had no activity to treat a certain condition. This product could be any therapeutic procedure that has an effect on the patient, symptom, syndrome or disease.^[3]^

In addition to the product considered to be placebo itself, there is what is called the “placebo effect”, this phenomenon occurs when a clinical result is obtained from the use of a product without an active ingredient. This phenomenon is still a mystery to modern medicine. This manifestation can be observed in several studies, mainly in those where symptoms such as pain are treated.^[4]^

Many authors explain the “placebo effect” associating it with psychological manifestations of the individual Placebo has shown during the years activity in order to modulate some responses to the active treatments in conditions as pain, some surgeries, anxiety, Parkinson disease. Thus, it is possible to verify the psychological factor that the placebo can have to the people who use this product.^[5]^

Although there is an improvement effect observed in some of the research participants with the use of placebo, it remains an inert product, without the addition of active principle, so its “action” is bound to psychological manifestations. Thus, the “placebo effect” does not justify the use placebo product as a comparator in a clinical study because it has no proven pharmacological activity that can interfere with the progression of the disease.^[6]^

However, the use of placebo in clinical studies is still a widely used methodological tool since they have a negative control function during medical research. Placebos are generally products with the same physical characteristics of medicines that will be tested and administered, without, however, the natural therapeutic effect thereof. The fact that this product is “identical” to the tested medicine is of fundamental importance to enable the study to be blinded.^[6]^

A comparison between the new and existing medicines or a placebo is imperative to assess the unique treatment benefits to the patient and knowledge of its risks.

Those individuals in the negative control group, that is, those to whom a placebo is administered, are treated as a mere object of research. This practice demonstrates that there is not the least concern for the health of those subjects who are willing to help in that clinical study for the development of a new treatment, contrary to all ethical issues outlined in Good Clinical Practice.^[7]^

Regarding the Declaration of Helsinki of the World Health Organization, the use of placebo is accepted in some situations. Except when there is no proven intervention, the use of placebo, or non-intervention, is acceptable; or when the use of placebo will not submit the subject to additional risks of serious or irreversible effects as a result of not receiving the best-proven intervention. Extreme care should be taken to avoid abuse of this option.^[8]^

Even with the ethical restriction, some studies are performed using placebo as the comparator. In this sense, justifications of placebo use must be discussed and only accepted if the patients will not be exposed to unnecessary risks. For some authors, the only ethical arguments for placebo use is the inexistence of comparator, and there was doubt about the benefits of placebo use in prophylaxis studies.^[9]^

The authors of some placebo-controlled trials do not uniformly agree that the use of placebo when a possible comparator is available is unethical. They believe that if patients are not harmed, such tests can ethically be carried out. Also, some studies with active control can present not reliable evidence of the effectiveness of a new therapy.^[10]^ In this sense, the focus should be on the benefits for the patients.

In this sense, it is essential to investigate if placebo use allows positive outcomes regarding efficacy and security compared to synthetic drug products. Besides, it is crucial to evaluate the relation of its use and pharmaceutical industry sponsorship and if the justification of use is considered ethically acceptable.

## 2 Methods

### 2.1 Protocol and registration

This review protocol was registered at the PROSPERO platform under the number CRD42018110829. This systematic review was conducted according to the Preferred Reporting Items for Systematic Reviews and Meta-analyses, following the Preferred Reporting Items for Systematic Reviews and Meta-Analyses Protocols checklist.^[11]^

The present study protocol is being reported in accordance with the reporting guidance provided in the Preferred Reporting Items for Systematic Reviews and Meta-Analyses Protocols statement^[12]^ (see Preferred Reporting Items for Systematic Reviews and Meta-Analyses Protocols checklist in Additional file 1).

### 2.2 Eligibility criteria

The acronym used for this question was PICO - participants, intervention, comparator, and outcomes.^[13]^ For this study, the population was the subjects of clinical trials, the intervention was the synthetic oral drug products, the comparison was placebo, and the outcomes were positive efficacy and safety tendencies when testing synthetic drug products.

Inclusion criteria: studies that had only 2 arms (placebo and synthetic drug) administered orally. Only phase III, randomized, double-blind studies were included in this systematic review. Primary outcome: The results of interest were drug efficacy, adverse effects, placebo effect, and ethical implications.

Exclusion criteria: studies published in non-Latin alphabet, clinical trials with medical devices and diagnostic methods, clinical trials with non-synthetic drug products, non-randomized or observational clinical trials, studies with only 1 intervention arm or more than 2 arms, clinical trials with non-oral medicines, sublingually, more than 1 clinical trial per article, studies that present medicines with other pharmaceutical forms than tablets and use of other medicines concomitant with placebo.

Studies that used concomitant medicines with placebo were included if the authors expressly mentioned that these medicines did not affect the outcome of the study.

### 2.3 Information sources and search strategy

PubMed, Cochrane, LILACS (BVS), Web of Science, Scopus, and Excerpta Medica dataBASE (EMBASE) databases will be searched. Gray literature will be identified through the databases Proquest (Dissertation and Theses), OpenGrey and Google Scholar. Headings (MeSH) will be used in order to ensure uniform search terms. The search strategy will be piloted to ensure sufficient specificity and sensitivity. A draft search strategy for PubMed/MEDLINE is provided in Complementary file 2 (PUBMED strategy;).

### 2.4 Screening and selection of studies

The Rayyan system was used to evaluate abstracts and remove duplicates.^[14]^ Randomized clinical trials that met all the inclusion criteria and passed the analysis of the exclusion criteria were evaluated in 2 phases. Phase 1: reading of all abstracts and selection of studies; phase 2: reading the full text and preparing the systematic review. Two independent reviewers (JA-S and CF) evaluated the quality of the trials and extracted the data following the standard Cochrane methodology, discrepancies resolved by a third participant (DM). Whenever necessary, they contacted the authors of the articles for additional information.

### 2.5 Data extraction

**Figure 1:**
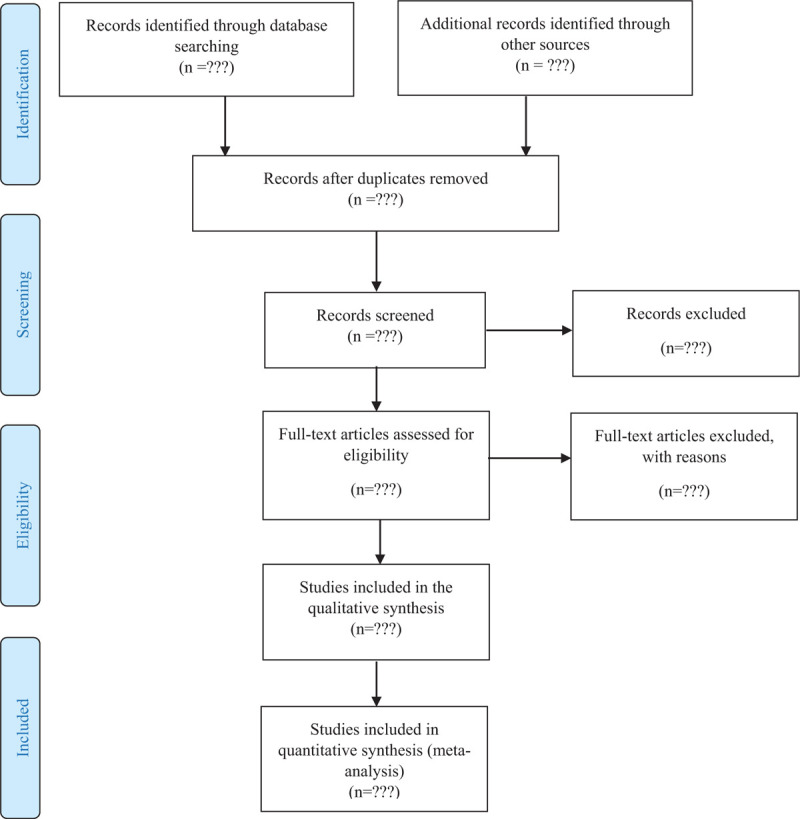
Flow diagram.

From each study, we extracted study design, arms used at the study, medicine or medicine class, disease, number of industry and non-industry funded articles, location, inclusion and exclusion criteria, and the primary purpose of the study. Since many studies have the design mentioned above, that is, the use of placebo as a comparator considering another pharmaceutical product, the intended analysis of this study design was stratified regarding all the inclusion criteria. The initial flowchart about this data extraction is present at Figure 1.

### 2.6 Evaluation of study quality

The analysis of the included clinical studies was performed according to the Cochrane bias risk assessment.^[15]^

### 2.7 Synthesis of included studies

The data will be aggregate considering the different placebo use strategies across the different types of sponsorship and the different justifications for this use. Quantitative positive results for oral medicines when comparing to the placebo will also be included and interpreted.

If data permits, the potential bias of publication, that is, the effect of the small sample studies and will be analyzed by the funnel chart and the Egger test.^[16,17]^

The heterogeneity between the studies will be assessed by inspection of the funnel plots for asymmetry and with Egger test, with small study effects when *P* values < .10. The heterogeneity between the studies was considered high if I^2^> 75%, moderate if 75% <I^2^ <25% and low if I^2^ <25%.^[18–21]^

## 3 Discussion

Regarding the design of clinical trials, it is clear that randomized controlled clinical trials (RCTs) are the gold standards for interventional results. There are some limitations, such as bureaucracy and high costs, to carry out these studies. However, it is undeniable that RCTs revolutionized medical research and improved the quality of healthcare, clarifying the benefits and disadvantages of numerous interventions, so only this type of study was included in this systematic review.^[9]^

Study designs that use the placebo as a control are also accepted in some cases, although it is essential to assess the reason behind its use. There was widespread adoption of the use of placebo in clinical trials after World War II. This type of design was chosen considering that a large number of new treatments were emerging at the time, so the use of placebos was readily accepted in relation to ethical rules. However, currently the use of placebo may raise some questions, mainly because it is possible to have another study design with less risk for the research subjects.^[22]^

The unethical use of placebo is a very critical issue that must be debated to avoid this type of design altogether. According to the Declaration of Helsinki: “In any medical study, all patients, including those in the control group, must be sure of the best proven diagnostic and therapeutic methods and no patient should suffer from unnecessary pain”.^[8]^

In this sense, if treatment for a given disease already exists, it is unethical to choose placebo as the control group, since the patient deserves to receive the best possible therapy. Considering the guideline published by the National Institutes of Health, with the theme Protecting Research Participants in Humans, some justifications for the use of the placebo were presented. These justifications are

1. When there are no approved and effective treatments for the condition.
2. If there is disagreement whether the standard treatment is better than the placebo.
3. When the additional risk represented by the use of placebo is lower and the suspension of current standard therapy would not lead to serious or permanent damage.
4. If the study is expected to result in generalized or significant benefits, and the receipt of placebo by individuals represents a minimal risk.^[23]^

Regarding the justification that there are no approved and effective treatments for the condition, for anti-cancer drugs trials, it is possible to justify the placebo use when no approved therapy exists or the available treatment has only minimal effects and/or presents serious adverse effects.^[24]^

In some cases, the physicians claimed that placebo might be quite effective and, frequently, produce less undesirable side effects. Therefore, the ethical problem with its use is when it is useless or even harmful, and when there is an alternative treatment. Placebo is considered nowadays not a pill absolutely inert; it can cause psychophysiological effects and benefits, mainly in pain, Parkinson disease, depression and emotion.^[25,26]^ These psychological effects can affect the results of clinical trials. Considering all this information, the researches should have a caution to choose placebo as the comparator in order to not bring risks to the subjects of the trial.

Lexchin et al (2003) treated about pharmaceutical industry sponsorship in clinical trials and research outcome and quality of the product of interest, it was concluded that research sponsored by the drug industry presented better results for the tested drug than studies funded by other sources. It shows that pharmaceutical industry intentions of having positive outcomes for their products, to it registered and available in the marketing, are more frequently reached when they sponsor the trial.^[27]^

Regarding all these critical points, it means; ethical issues, pharmaceutical industry interests, justification of placebo use, marketing authorization approval for regulatory authorities, positive results for new drug products; this systematic review was developed to try to link them and give a robust effect about this scenario. In this sense, it is important to evaluate the use of placebo at RCTs designs and its justification. Besides, the role of pharmaceutical industry sponsorship relation to it.

## Author contributions

**Investigation:** Juliana C. R. Alves da Silva, Cecília Menezes Farinasso.

**Methodology:** Juliana C. R. Alves da Silva, Cecília Menezes Farinasso, Cristine Stefani, Patrícia Medeiros de Souza, Grasiela Piuvezam, Helaine Carneiro Capucho.

**Project administration:** Grasiela Piuvezam, Juliana C. R. Alves da Silva, Kesley Pablo Morais de Azevedo, Helaine Carneiro Capucho.

**Reading and Final Revision of the Text:** All.

**Research for the articles:** Juliana C. R. Alves da Silva and Cecília Menezes Farinasso.

**Writing – original draft:** Juliana C. R. Alves da Silva, Ana Cláudia Morais Godoy Figueiredo.

**Writing – review & editing:** Juliana C. R. Alves da Silva, Cecília Menezes Farinasso, Dayde Lane Mendonça da Silva, Cristine Stefani, Ana Cláudia Morais Godoy Figueiredo, Patrícia Medeiros de Souza, Grasiela Piuvezam, Helaine Carneiro Capucho.

**Writing of the scientific paper:** Juliana C. R. Alves da Silva, Cecília Menezes Farinasso, Ana Cláudia Morais Godoy Figueiredo, Dayde Lane Mendonça da Silva, Helaine Carneiro Capucho e Grasiela Piuvezam.

## Supplementary Material

Supplemental Digital Content
