# Supplementary material for: Placebo use and outcome quality: A protocol for systematic review and meta-analysis
Source: Medicine (Baltimore). 2020 Oct 30;99(44):e22915. doi: 10.1097/MD.0000000000022915 (PMC7598826; doi:10.1097/MD.0000000000022915)
Supplement: Supplemental Digital Content [file medi-99-e22915-s001.docx]

| **Additional file 2: Search strategy for PubMed/MEDLINE** | ((((("randomized controlled trial"[pt] OR "controlled clinical trial"[pt] OR "randomized"[tiab] OR "clinical trials as topic"[mesh:noexp] OR "randomly"[tiab] OR "trial"[ti]) NOT ("animals"[mh] NOT "humans"[mh])) AND ("Treatment Outcome"[All Fields] OR "Outcome, Treatment"[All Fields] OR "Clinical Effectiveness"[All Fields] AND ("treatment outcome"[MeSH Terms] OR ("treatment"[All Fields] AND "outcome"[All Fields]) OR "treatment outcome"[All Fields] OR ("clinical"[All Fields] AND "effectivenesses"[All Fields])) OR "Effectiveness, Clinical"[All Fields] OR ("treatment outcome"[MeSH Terms] OR ("treatment"[All Fields] AND "outcome"[All Fields]) OR "treatment outcome"[All Fields] OR ("effectivenesses"[All Fields] AND "clinical"[All Fields])) OR "Patient-Relevant Outcome"[All Fields] OR ("treatment outcome"[MeSH Terms] OR ("treatment"[All Fields] AND "outcome"[All Fields]) OR "treatment outcome"[All Fields] OR ("outcome"[All Fields] AND "patient"[All Fields] AND "relevant"[All Fields])) OR ("treatment outcome"[MeSH Terms] OR ("treatment"[All Fields] AND "outcome"[All Fields]) OR "treatment outcome"[All Fields] OR ("outcomes"[All Fields] AND "patient"[All Fields] AND "relevant"[All Fields])) OR "Patient Relevant Outcome"[All Fields] OR "Patient-Relevant Outcomes"[All Fields] OR "Clinical Efficacy"[All Fields] OR "Efficacy, Clinical"[All Fields] OR "Treatment Effectiveness"[All Fields] OR "Effectiveness, Treatment"[All Fields] OR "Treatment Efficacy"[All Fields] OR "Efficacy, Treatment"[All Fields] OR "Rehabilitation Outcome"[All Fields] OR "Outcome, Rehabilitation"[All Fields] AND "Drug Related Side Effects and Adverse Reactions"[All Fields] OR "Side Effects of Drugs"[All Fields] OR "Drug Side Effects"[All Fields] OR "Drug Side Effect"[All Fields] OR ("drug-related side effects and adverse reactions"[MeSH Terms] OR ("drug-related"[All Fields] AND "side"[All Fields] AND "effects"[All Fields] AND "adverse"[All Fields] AND "reactions"[All Fields]) OR "drug-related side effects and adverse reactions"[All Fields] OR ("effects"[All Fields] AND "drug"[All Fields] AND "side"[All Fields])) OR ("drug-related side effects and adverse reactions"[MeSH Terms] OR ("drug-related"[All Fields] AND "side"[All Fields] AND "effects"[All Fields] AND "adverse"[All Fields] AND "reactions"[All Fields]) OR "drug-related side effects and adverse reactions"[All Fields] OR ("side"[All Fields] AND "effect"[All Fields] AND "drug"[All Fields])) OR "Side Effects, Drug"[All Fields])) AND ("Adverse Drug Reaction"[All Fields] OR "Adverse Drug Reactions"[All Fields] OR "Drug Reaction, Adverse"[All Fields] OR "Drug Reactions, Adverse"[All Fields] OR ("drug-related side effects and adverse reactions"[MeSH Terms] OR ("drug-related"[All Fields] AND "side"[All Fields] AND "effects"[All Fields] AND "adverse"[All Fields] AND "reactions"[All Fields]) OR "drug-related side effects and adverse reactions"[All Fields] OR ("reactions"[All Fields] AND "adverse"[All Fields] AND "drug"[All Fields])) OR "Adverse Drug Event"[All Fields] OR "Adverse Drug Events"[All Fields] OR ("drug-related side effects and adverse reactions"[MeSH Terms] OR ("drug-related"[All Fields] AND "side"[All Fields] AND "effects"[All Fields] AND "adverse"[All Fields] AND "reactions"[All Fields]) OR "drug-related side effects and adverse reactions"[All Fields] OR ("drug"[All Fields] AND "event"[All Fields] AND "adverse"[All Fields])) OR ("drug-related side effects and adverse reactions"[MeSH Terms] OR ("drug-related"[All Fields] AND "side"[All Fields] AND "effects"[All Fields] AND "adverse"[All Fields] AND "reactions"[All Fields]) OR "drug-related side effects and adverse reactions"[All Fields] OR ("drug"[All Fields] AND "events"[All Fields] AND "adverse"[All Fields])) OR "Drug Toxicity"[All Fields] OR "Toxicity, Drug"[All Fields] OR "Drug Toxicities"[All Fields] OR ("drug-related side effects and adverse reactions"[MeSH Terms] OR ("drug-related"[All Fields] AND "side"[All Fields] AND "effects"[All Fields] AND "adverse"[All Fields] AND "reactions"[All Fields]) OR "drug-related side effects and adverse reactions"[All Fields] OR ("toxicities"[All Fields] AND "drug"[All Fields])))) AND ("medicine"[MeSH Terms] OR "medicine"[All Fields])) AND "placebo"[tiab] |
| --- | --- |
